# C-EBPβ mediates in cigarette/IL-17A-induced bronchial epithelial–mesenchymal transition in COPD mice

**DOI:** 10.1186/s12890-021-01738-6

**Published:** 2021-11-18

**Authors:** Shuyuan Chu, Libing Ma, Yashan Wu, Xiaoli Zhao, Bo Xiao, Qilu Pan

**Affiliations:** 1 Laboratory of Respiratory Disease, The Affiliated Hospital of Guilin Medical University, Guilin, 541001 Guangxi China; 2 Department of Respiratory and Critical Care Medicine, The Affiliated Hospital of Guilin Medical University, Guilin, 541001 Guangxi China; 3 Guangxi University of Chinese Medicine, Nanning, 530222 Guangxi China

## Abstract

**Background:**

Cigarettes smoking and IL-17A contribute to chronic obstructive pulmonary disease (COPD), and have synergistical effect on bronchial epithelial cell proliferation. CCAAT/enhancer-binding protein β (C-EBPβ) could be induced by IL-17A and is up-regulated in COPD. We explored the effect of cigarettes and IL-17 on bronchial epithelial–mesenchymal transition (EMT) in COPD mice and potential mechanism involved with C-EBPβ in this study.

**Methods:**

COPD model was established with mice by exposing to cigarettes. E-Cadherin, Vimentin, IL-17A and C-EBPβ distributions were detected in lung tissues. Primary bronchial epithelial cells were separated from health mice and cocultured with cigarette smoke extract (CSE) or/and IL-17A. E-Cadherin, Vimentin and IL-17 receptor (IL-17R) expressions in vitro were assessed. When C-EBPβ were silenced by siRNA in cells, E-Cadherin, Vimentin and C-EBPβ expressions were detected.

**Results:**

E-Cadherin distribution was less and Vimentin distribution was more in bronchus of COPD mice than controls. IL-17A and C-EBPβ expressions were higher in lung tissues of COPD mice than controls. In vitro, C-EBPβ protein expression was highest in CSE + IL-17A group, followed by CSE and IL-17A groups. E-cadherin expression in vitro was lowest and Vimentin expression was highest in CSE + IL-17A group, followed by CSE or IL-17A group. Those could be inhibited by C-EBPβ silenced.

**Conclusions:**

C-EBPβ mediates in cigarette/IL-17A-induced bronchial EMT in COPD mice. Our findings contribute to a better understanding on the progress from COPD to lung cancers, which will provide novel avenues in preventing tumorigenesis of airway in the context of cigarette smoking.

## Introduction

Patients with COPD are shown an increase risk of lung cancer [1]. Epithelial–mesenchymal transition (EMT) in airway was found in COPD, which plays an important role in tumorigenesis [2, 3]. Cigarette smoking, the obvious culprit linking COPD and lung cancer [4], could induce epithelial–mesenchymal transition (EMT) in airway [5]. Previous study demonstrated that cigarette smoke extract (CSE) could stimulate EMT in human pulmonary cells A549 and human bronchial cells BEAS2B, which was independent to TGF-β signaling [5]. Those findings suggest that in response to CSE exposure, human airway cells undergo a functional phenotypic shift, namely EMT, which contribute to increased collagen deposition in COPD lungs [5]. Thus, we investigated the influence of cigarette smoking on bronchial EMT in COPD mice in the present study, which may contribute to further understanding on the mechanism of interaction between COPD and lung cancer.

Interestingly, IL-17A could promote both the development of COPD and lung cancer [6, 7]. IL-17A could not only induce production of chemokine and cytokines from bronchial epithelial cells, but also promote airway remodeling and EMT [8–11]. IL-17A is a crucial component of immune microenvironment, in which human A549 cells could be induced EMT and migration [12]. Previous study found that CSE could enhance IL-17 receptor (IL17R) expression in human bronchial epithelial cell line 16HBE, and moreover could synergistically work with IL-17A to induce proliferation of 16HBE [13]. Those findings indicate a synergistical effect of CSE and IL-17A on bronchial epithelial cell. Since CSE could induce EMT of bronchial epithelial cells in vitro and in vivo [14], we explored the effect of CSE and IL-17A on bronchial EMT in COPD mice model in this study.

EMT of several kinds of epithelial cells, such as mammary, gastric and pancreatic cells, could be regulated by CCAAT/enhancer-binding protein β (C-EBPβ) [15–17]. C-EBPβ is a member of CCAAT/enhancer-binding protein family, which is one of transcription factors of the basic-leucine zipper class. It’s implicated in various cellular events including cell energy metabolism, cell proliferation and differentiation [18]. C-EBPβ also plays a critical role in inflammation [19]. That may be because promoters of many pro-inflammatory genes contain putative C/EBPβ consensus sequences [20]. In airway, C-EBPβ plays a role in determining airway epithelial differentiation [21]. Moreover, in COPD pathogenesis, C-EBPβ may influence long-term respiratory outcomes by regulating host defenses and inflammatory response to cigarette smoke [22]. In advanced COPD, C-EBPβ expression in small airway epithelia was significantly increased, and exerts pro-inflammatory effects in the context of cigarette smoking [23]. Interestingly, C-EBPβ expression could be induced by IL-17A via IL-17R [24]. Therefore, we further investigated C-EBPβ signaling as potential mechanism about the effect of CSE and IL-17 on bronchial EMT in vitro in this study.

## Methods

### Animals

Eight-week-old male C57BL/6 mice were purchased from Shanghai Laboratory Animal Company, (Shanghai, China). The mice model was established as previously described [25]. Briefly, ten mice in COPD group were exposed to five cigarettes (Nanning Jiatianxia unfiltered cigarettes, 12 mg of tar and 0.9 mg of nicotine) four times per day with 30 min smoke-free intervals in a closed 0.75-m^3^ room, 5 days per week for up to 12 or 24 weeks. Mice tolerated CS (cigarette smoking) exposure without evidence of toxicity (carboxyhemoglobin levels ∼10% and no weight loss). An optimal smoke:air ratio of 1:6 was obtained. Ten mice in control group were exposed to air. All experiments were repeated three times.

Animal experiments were approved by the Institutional Animal Care and Use Committee of Guilin Medical University and conform to National Institutes of Health guidelines for the use of rodents. This study was reported in accordance with ARRIVE guidelines.

### Histology, immunohistochemistry and immunofluorescence staining for lung tissues

Formalin-fixed and paraffin-embedded lung tissues of mice were cut in sections, which were stained with hematoxylin and eosin (HE), Periodic acid–Schiff (PAS) and MASSON to respectively evaluate airway inflammation, goblet cell hyperplasia and mucous secretion, and extracellular matrix in the lung tissues [26].

For immunohistochemistry, lung-tissue slides were incubated with primary rabbit anti-mouse antibody against α-SMA (dilution 1:200, ab5694; Abcam, Cambridge, UK), C-EBPβ (1:200, ab53138; Abcam, Cambridge, UK), or rat anti-mouse antibody against IL-17 (1:200, ab118869; Abcam, Cambridge, UK) for overnight at 4 °C, and then were incubated for 30 min at 37 °C each with horseradish peroxidase conjugated anti-rabbit or anti-rat IgG antibody and subsequent rinses in PBS three times for 5 min. 3′3-diaminobenzidine-tetrahydrochloride was applied as a chromogen for 1–5 min. Sections were counterstained in haematoxylin for 5–10 min.

For immunofluorescence staining, lung-tissue slides were incubated with primary mouse anti-mouse antibody against E-cadherin (1:100, ab76055; Abcam, Cambridge, UK), or rabbit anti-mouse antibody against Vimentin (1:100, ab92547; Abcam, Cambridge, UK) at 4 °C overnight, and then were incubated with Alexa Fluor 594-conjugated Goat Anti-Mouse IgG(H + L)(SA00006-3, Proteintech, IL, USA) or Alexa Fluor 594-conjugated Goat Anti-Rabbit IgG(H + L)(SA00006-4, Proteintech, IL, USA). Finally, slides were staining with DAPI (4′,6-diamidino-2-phenylindole) at 37 °C for 10 min. Micrographs were obtained using a microscope (BA210T, motic, Xiamen, China).

### Cell culture

Health mice were anaesthetized using 2% isoflurane inhalation and killed by cervical dislocation. The bronchus was isolated from lobes of lung, minced to small pieces and digested by 0.05% pronase (Sigma, MA, USA) in DMEM/F12 media (Invitrogen, CA, USA) at 4 °C overnight. Digestion was stopped by adding FBS (Gibco, CA, USA). The bronchial epithelial cells were identified by CK-18 immunofluorescence staining.

Murine bronchial epithelial cells were cultured for 24 h, and then were cocultured with cigarette smoke extract (CSE) or/and IL-17A. CSE was prepared as previously reported [27]. Briefly, filtered cigarettes (Nanning Jiatianxia unfiltered cigarettes, Guangxi, China) were smoked using a peristaltic pump (VWR International) after cutting the filters. Each cigarette was smoked with a 1.5 cm butt remaining. Four cigarettes were bubbled through 40 ml of cell growth medium, and this solution, regarded as 100% strength CSE. In CSE group, bronchial epithelial cells were induced by 20% CSE. In IL-17A group, the cells were induced by 50 ng/ml IL-17A (Biolegend, CA, USA). In CSE + IL-17A group, the cells were cocultured with 20% CSE and 50 ng/ml IL-17A (Biolegend, CA, USA). Cells in all groups were cultured at 37 °C with 5%CO_2_ for 72 h. The cells without administration of CSE or IL-17A were control group.

### Transfection of small interfering RNA (siRNA)

The C-EBPβ siRNA and scramble siRNA were synthesized by Ribobio (Guangzhou, China). The siRNAs were transfected with riboFECTTM CP Reagent according to the manufacturer’s protocol. Bronchial epithelial cells WT mice were transiently transfected with 20 nM scramble or siRNA. After transfection for 24 h at 37 °C in 5% CO_2_, the cells were prepared as IL-17A group, CSE group, CSE + IL-17A group and controls as described above. The inhibition of C-EBPβ was identified by western blotting.

### Quantitative real‑time PCR

Total RNA from lung tissues and cells were respectively extracted using the TRIzol reagent (Invitrogen, CA, USA) according to the manufacturer’s protocol, and RNA was reverse transcribed into cDNA (Fermentas, Ontario, Canada). A quantitative real-time polymerase chain reaction (PCR) was performed with PIKO REAL 96 instrument (Thermo, MA, USA) and the SYBGREEN PCR Mix Mix (Applied Biosystems, CA, USA) in accordance with the manufacturer’s protocol. Each set of experiments was repeated three times. The 30 μl PCR reactions (with 2 μl cDNA, 0.5 μl (10 μM) forward and 0.5 μl (10 μM) reverse primers, 15 μl SYBR green, and 12 μl PCR H2O) were undergone 10 min at 95 °C, then 40 cycles of 15 s at 95 °C and 60 s at 60 °C. The primers were as follows: mouse E-Cadherin F, 5′-GCAGTTCTGCCAGAGAAACC-3′, and R, 5′-TGGATCCAAGATGGTGATGA-3′; mouse Vimentin F, 5′-GCCAACCTTTTCTTCCCTGA-3′, and R, 5′-TCAAGGTCATCGTGATGCTG-3′; mouse β-actin F, 5′-GATCTGGCACCACACCTTCT-3′, and R, 5′-CTTTTCACGGTTGGCCTTAG-3′. The level of mRNA expression was reported as fold change using the 2^−ΔΔCt^ method.

### Western blot analysis for cells

The bronchial epithelial cells were treated with 200 μl RIPA for 10 min on ice, and then were centrifuged at 12,000×*g* (4 °C) for 15 min. The loaded proteins (50–170 μg) were separated on a 10% SDS-PAGE, followed by transferring onto PVDF membranes. The samples were blocked with TBS-Tween 20 (TBST) containing 5% skim milk for 60 min at room temperature, and at 4 °C overnight, then 30 min at room temperature. The membranes were incubated with rabbit anti-mouse antibody against mouse C-EBPβ (1:1000, ab53138; Abcam, Cambridge, UK), and mouse anti-mouse antibody against β-actin (1:5000, 60008-1-Ig; Proteintech, IL, USA) for 90 min at room temperature, and then they were incubated with horseradish peroxidase conjugated goat anti-rabbit (1:6000, SA00001-2; Proteintech, IL, USA) or anti-mouse antibody (1:5000, SA00001-1; Proteintech, IL, USA) for 90 min at room temperature. At last, blots were developed with the ECL Plus reagents (Thermo pierce, IL, USA).

### Immunohistochemistry and immunofluorescence staining for cells

For immunohistochemistry, the slides of cells were incubated with primary rabbit anti-mouse antibody against IL-17R (1:50, ab180904; Abcam, Cambridge, UK) at 4 °C overnight, and then were incubated with horseradish peroxidase conjugated goat anti-rabbit IgG antibody (PV-9000, Zisbio, Beijing, China) at 37 °C for 30 min. After rinsing with PBS for three times, 3′3-diaminobenzidine-tetrahydrochloride was applied on the slides as a chromogen for 1–5 min. Slides were counterstained in haematoxylin for 5–10 min.

For immunofluorescence, the slides of cells were incubated with primary mouse anti-mouse antibody against Cytokeratin18 (1:50, ab668; Abcam, Cambridge, UK), mouse anti-mouse antibody against E-cadherin (1:50, ab76055; Abcam, Cambridge, UK), or rabbit anti-mouse antibody against Vimentin (1:50, ab92547; Abcam, Cambridge, UK) at 4 °C overnight, and then were incubated with Alexa Fluor 594-conjugated Goat Anti-Mouse IgG(H + L)(SA00006-3, Proteintech, IL, USA) or Alexa Fluor 594-conjugated Goat Anti-Rabbit IgG(H + L)(SA00006-4, Proteintech, IL, USA). Finally, slides were stained with DAPI (4′,6-diamidino-2-phenylindole) at 37 °C for 10 min.

### Statistical analysis

Group data were expressed as the mean ± standard deviation (SD). Significant differences were evaluated using t-test. *P* values < 0.05 were considered to be statistically significant. Statistical analyses were performed using SPSS 21.0 (IBM SPSS Inc., Chicago, IL, USA).

## Results

### Cigarettes smoking induces airway inflammation and airway remolding of COPD mice

**Fig. 1:**
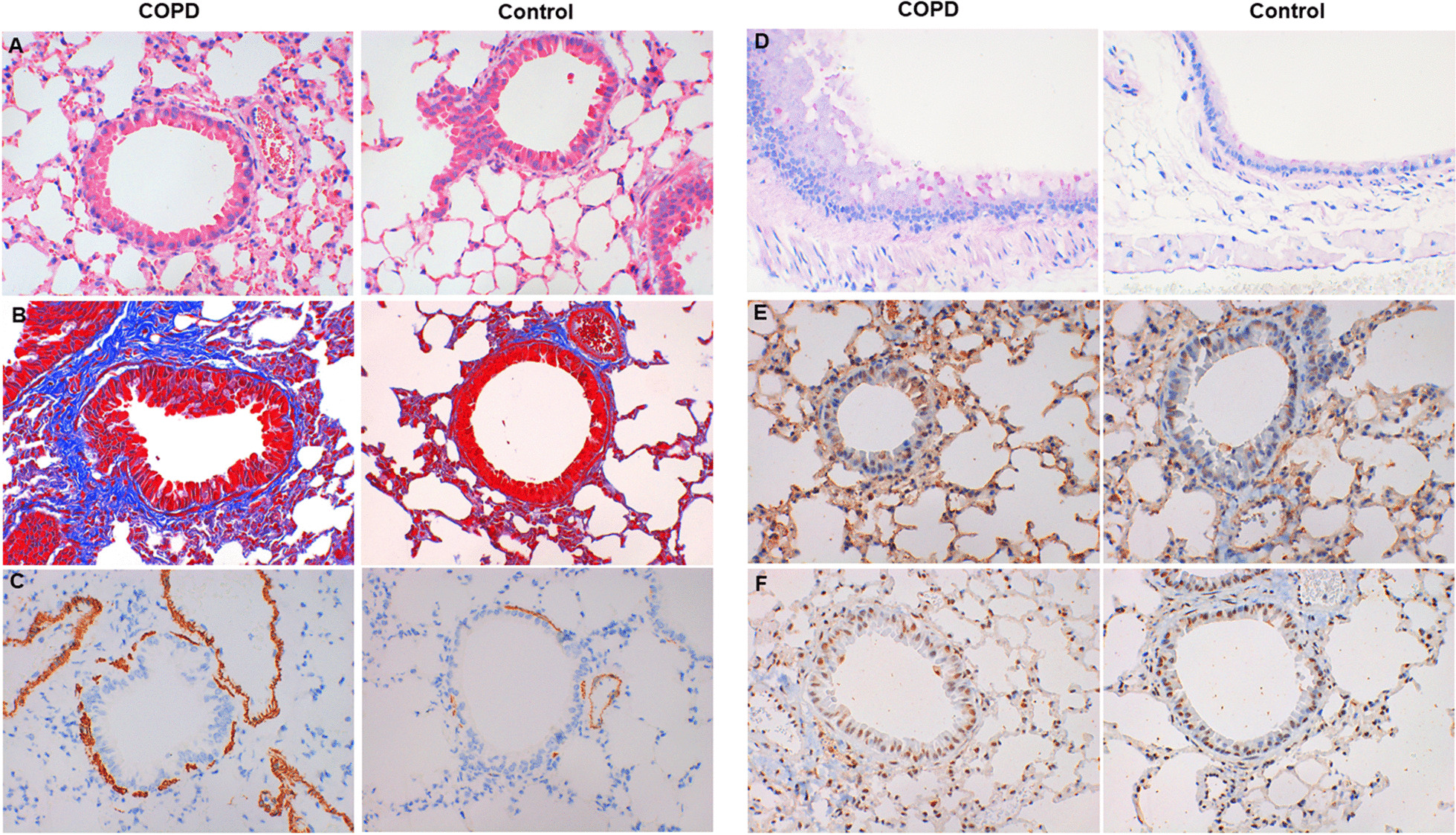
Histology and Immunohistochemistry of lung tissues. **a** HE staining. **b** MASSON staining. **c** α-SMA staining. **d** PSA staining. **e** IL-17A+ cells. **f** C-EBPβ+ cells. (× 400 magnification)

In lung tissues, COPD mice had an increased inflammation (Fig. 1a), extracellular matrix (Fig. 1b), smooth muscle thickening (Fig. 1c), goblet cell hyperplasia (Fig. 1d) and mucous secretion (Fig. 1d) when compared with controls. The mean linear intercept (MLI) and destructive index in COPD mice were larger than that in controls (MLI: COPD vs. controls = 74.58 ± 4.81 vs. 64.92 ± 5.40, *P* = 0.001; destructive index: COPD vs. controls = 36.37 ± 6.94 vs. 27.24 ± 5.54, *P* = 0.004).

In lung tissues, COPD mice had an increased expression of IL-17A (Fig. 1e) and C-EBPβ (Fig. 1f) compared with controls.

### Cigarettes smoking induces airway EMT of COPD mice

**Fig. 2:**
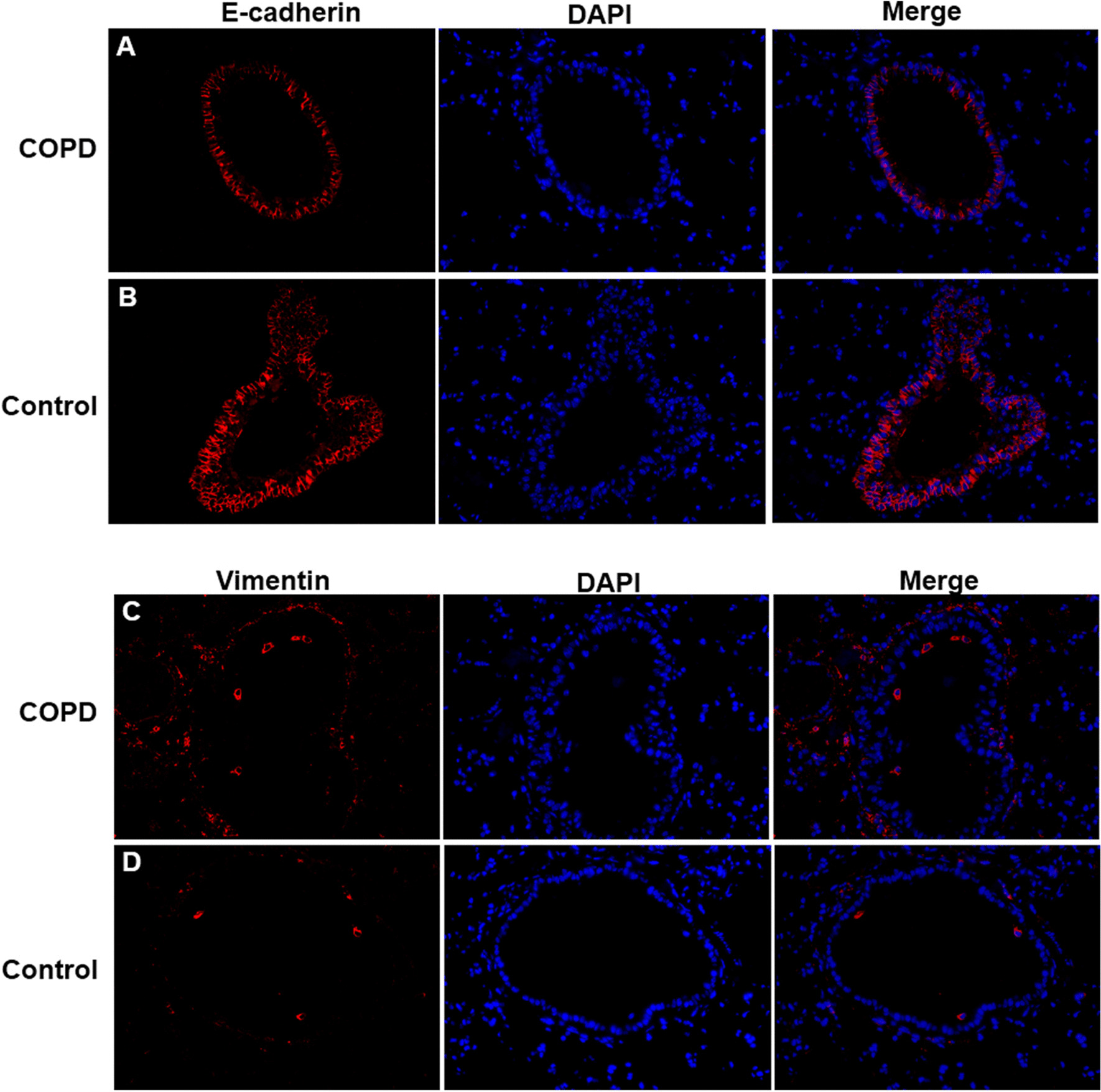
Immunohistochemistry of E-cadherin and Vimentin in lung tissues. **a** COPD group: E-Cadherin. **b** Control group: E-Cadherin. **c** COPD group: Vimentin. **d** Control group: Vimentin. (×400 magnification)

**Fig. 3:**
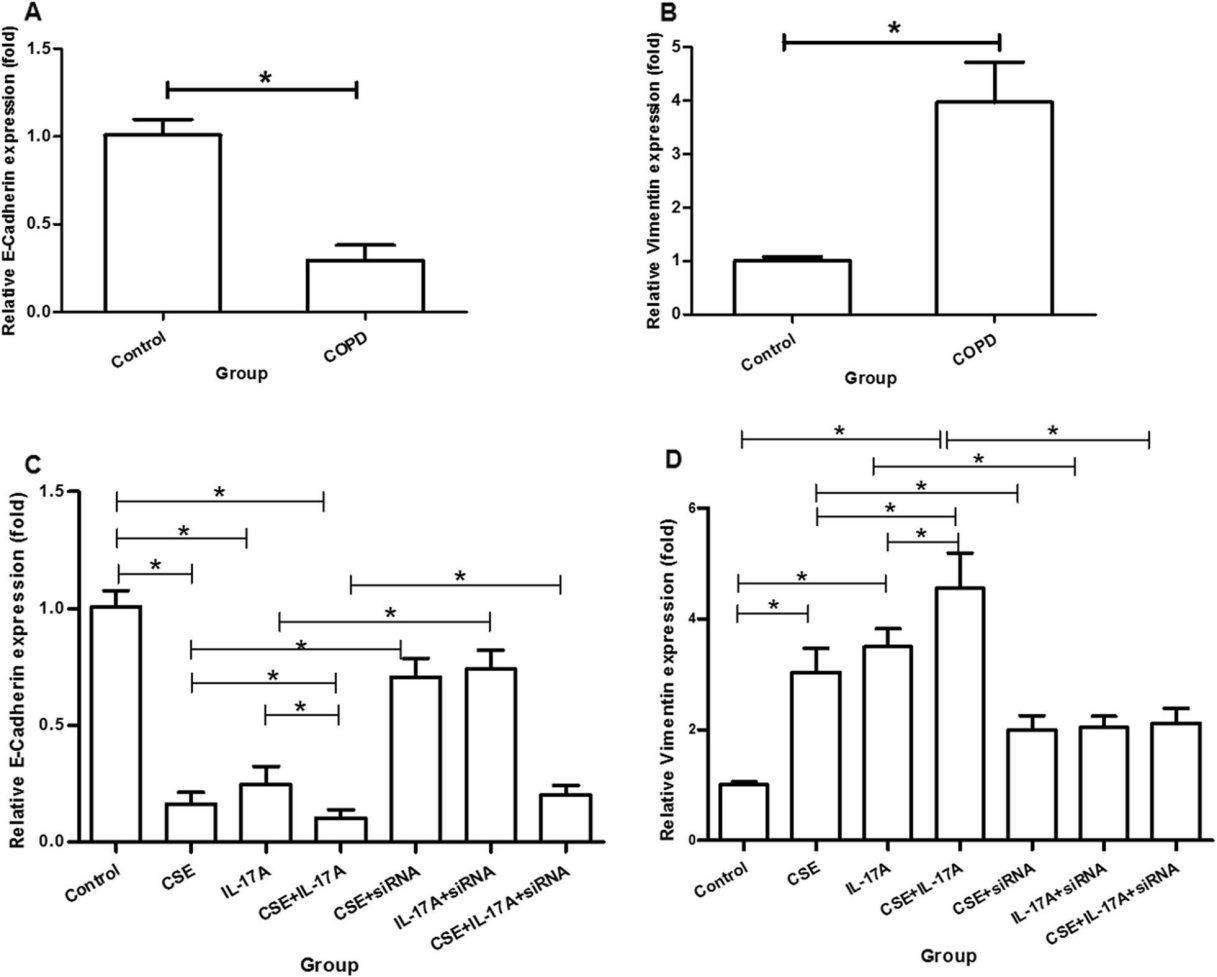
E-cadherin and Vimentin mRNA expressions. **a** E-Cadherin expression in lung tissues. **b** Vimentin expression in lung tissues. **c** E-Cadherin expression in bronchial epithelial cells. **d** Vimentin expression in bronchial epithelial cells. *P < 0.05

Immunofluorescence staining showed that E-Cadherin distribution (Fig. 2a) was decreased and Vimentin distribution (Fig. 2c) was increased in bronchus of COPD group when compared with controls (Fig. 2b, d). The mRNA expressions of E-Cadherin and Vimentin in lung tissues were in consistent with the protein distributions from immunofluorescence staining (Fig. 3a, b).

### Cigarette and IL-17A synergistically induces IL-17R expression in bronchial epithelial cells

**Fig. 4:**
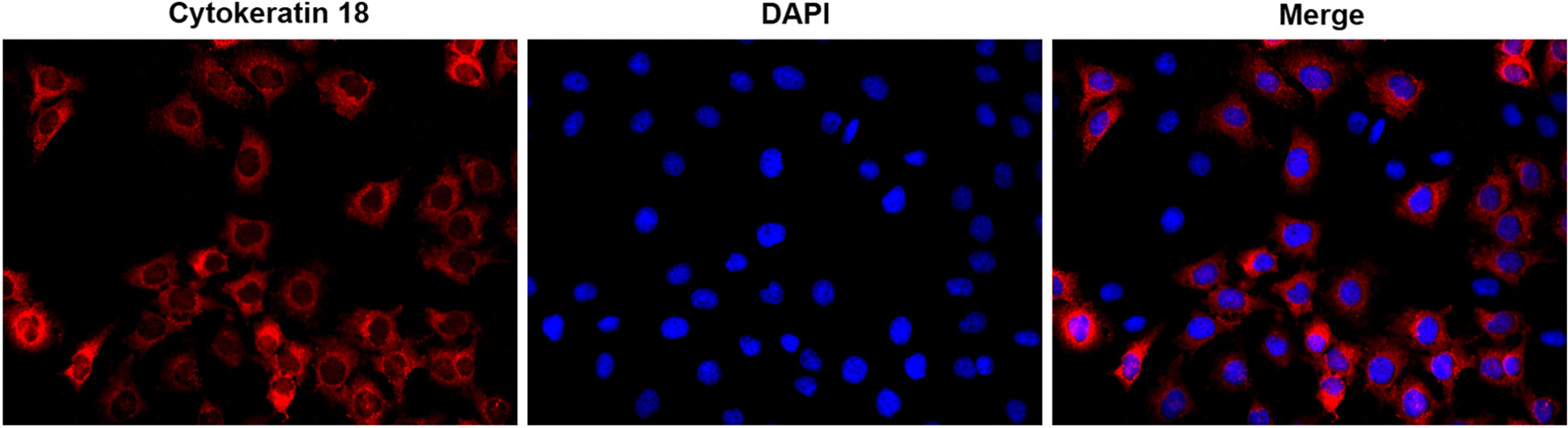
Cells identification by immunofluorescence staining of CK-18. (× 400 magnification)

The primary murine bronchial epithelial cells were identified by immunofluorescence staining of Cytokeratin18 (Fig. 4).

**Fig. 5:**
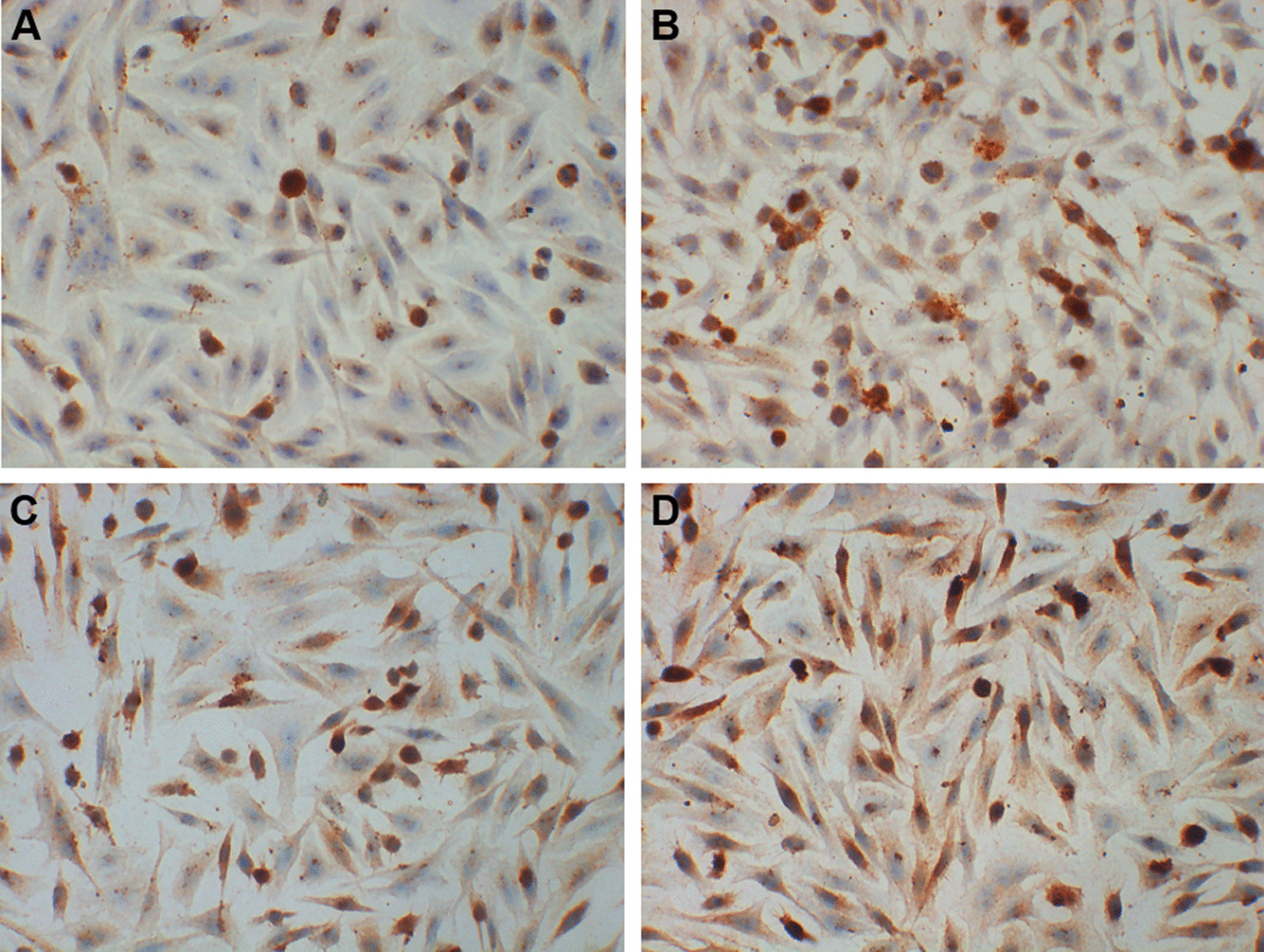
Immunohistochemistry staining of IL-17R in bronchial epithelial cells. **a** Control group. **b** CSE group. **c** IL-17A group. **d** CSE + IL-17A group. (× 400 magnification)

The expression of IL-17R in bronchial epithelial cells was higher in CSE group and IL-17A group than controls. It’s even higher in CSE + IL-17A group than CSE group or IL-17A group (Fig. 5). These results suggest that CSE or IL-17A could induce IL-17R expression in bronchial epithelial cells. And CSE plays a synergistical role with IL-17A in inducing the IL-17R expression.

### Cigarette and IL-17A synergistically induces epithelial–mesenchymal transition of bronchial epithelial cells

**Fig. 6:**
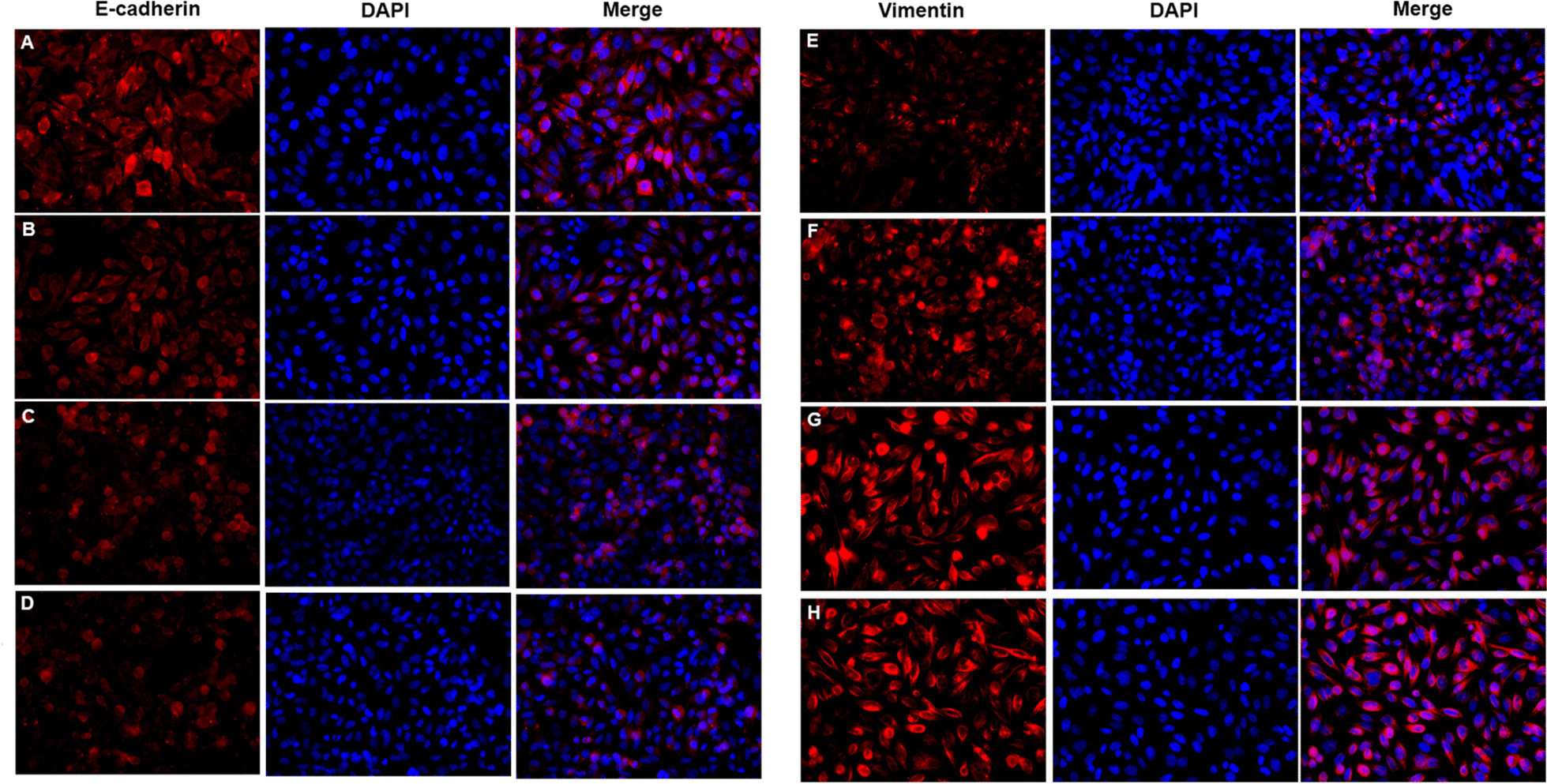
Immunofluorescence staining of E-cadherin and Vimentin in bronchial epithelial cells. **a** Control group: E-cadherin. **b** CSE group: E-cadherin. **c** IL-17A group: E-cadherin. **d** CSE + IL-17A group: E-cadherin. **e** Control group: Vimentin. **f** CSE group: Vimentin. **g** IL-17A group: Vimentin. **h** CSE + IL-17A group: Vimentin. (× 400 magnification)

The protein expression of E-cadherin in bronchial epithelial cells was decreased in CSE group (Fig. 6b) and IL-17A group (Fig. 6c), when compared with controls (Fig. 6a). E-cadherin expression was lower in CSE + IL-17A group (Fig. 6d) than CSE group or IL-17A group. In contrast, the protein expression of Vimentin in bronchial epithelial cells was enhanced in CSE group (Fig. 6f) and IL-17A group (Fig. 6g) compared with controls (Fig. 6e). The Vimentin expression was higher in CSE + IL-17A group (Fig. 6h) than CSE group or IL-17A group. The mRNA expressions of E-Cadherin and Vimentin in bronchial epithelial cells were the same as the protein expressions (Fig. 3c, d). These results indicate that CSE or IL-17A could induce bronchial EMT, and both of CSE and IL-17A synergistically promote the bronchial EMT.

### C-EBPβ is activated by CSE and IL-17A

**Fig. 7:**
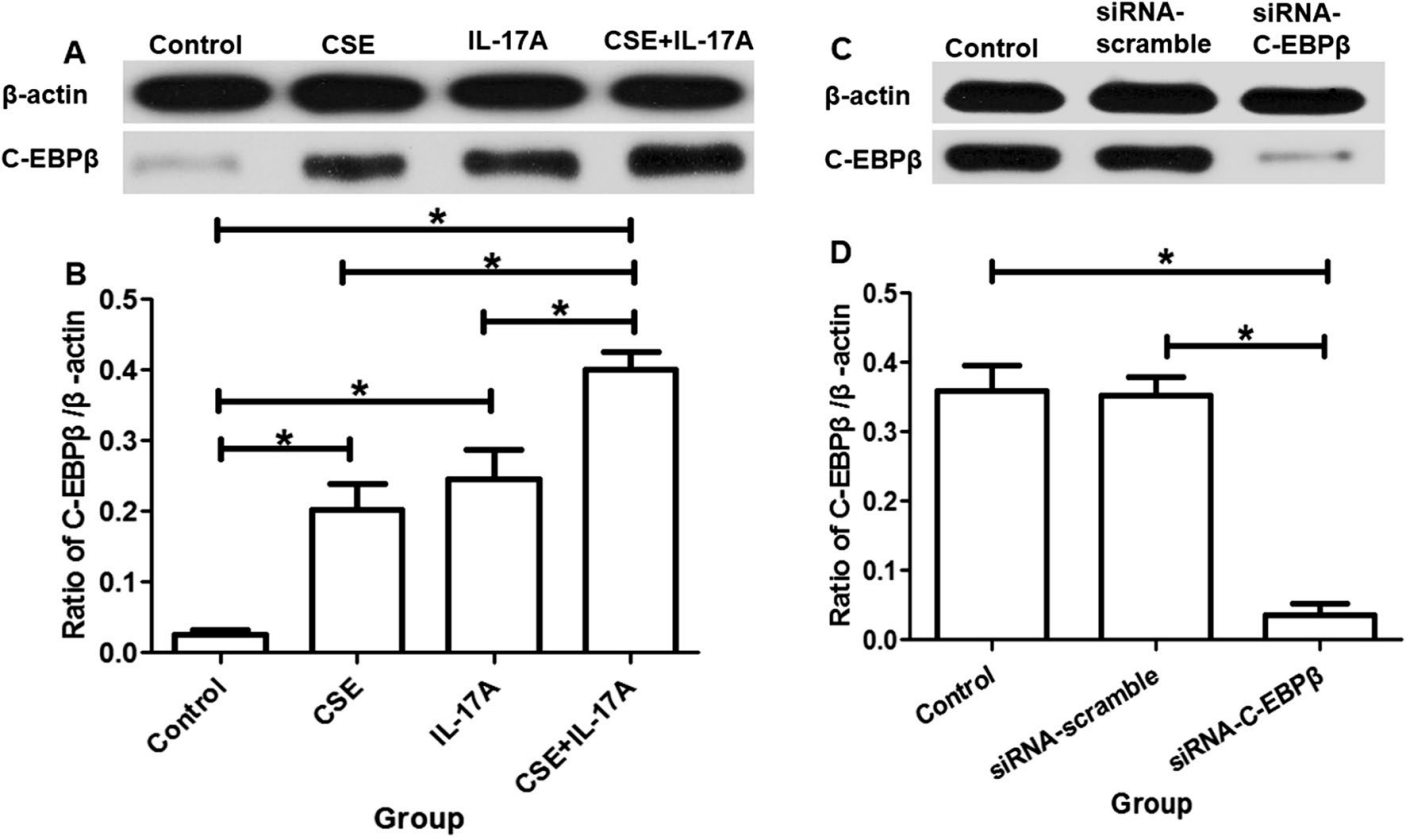
The protein expression of C-EBPβ in bronchial epithelial cells. **a** and **b** C-EBPβ expression without C-EBPβ-siRNA interfered. **c** and **d** C-EBPβ protein expression after C-EBPβ-siRNA interfered. *P < 0.05

The protein expression of C-EBPβ in bronchial epithelial cells was higher in CSE group and IL-17A group than controls. It’s higher in CSE + IL-17A group than CSE group or IL-17A group (Fig. 7a, b). This result suggests that C-EBPβ is up-regulated by CSE or IL-17A. CSE could coordinate with IL-17A in C-EBPβ up-regulation. When C-EBPβ siRNA was transfected into the cells, C-EBPβ protein expression was significantly reduced (Fig. 7c, d).

### C-EBPβ mediates in CSE/IL-17A-induced epithelial–mesenchymal transition of bronchial epithelial cells

**Fig. 8:**
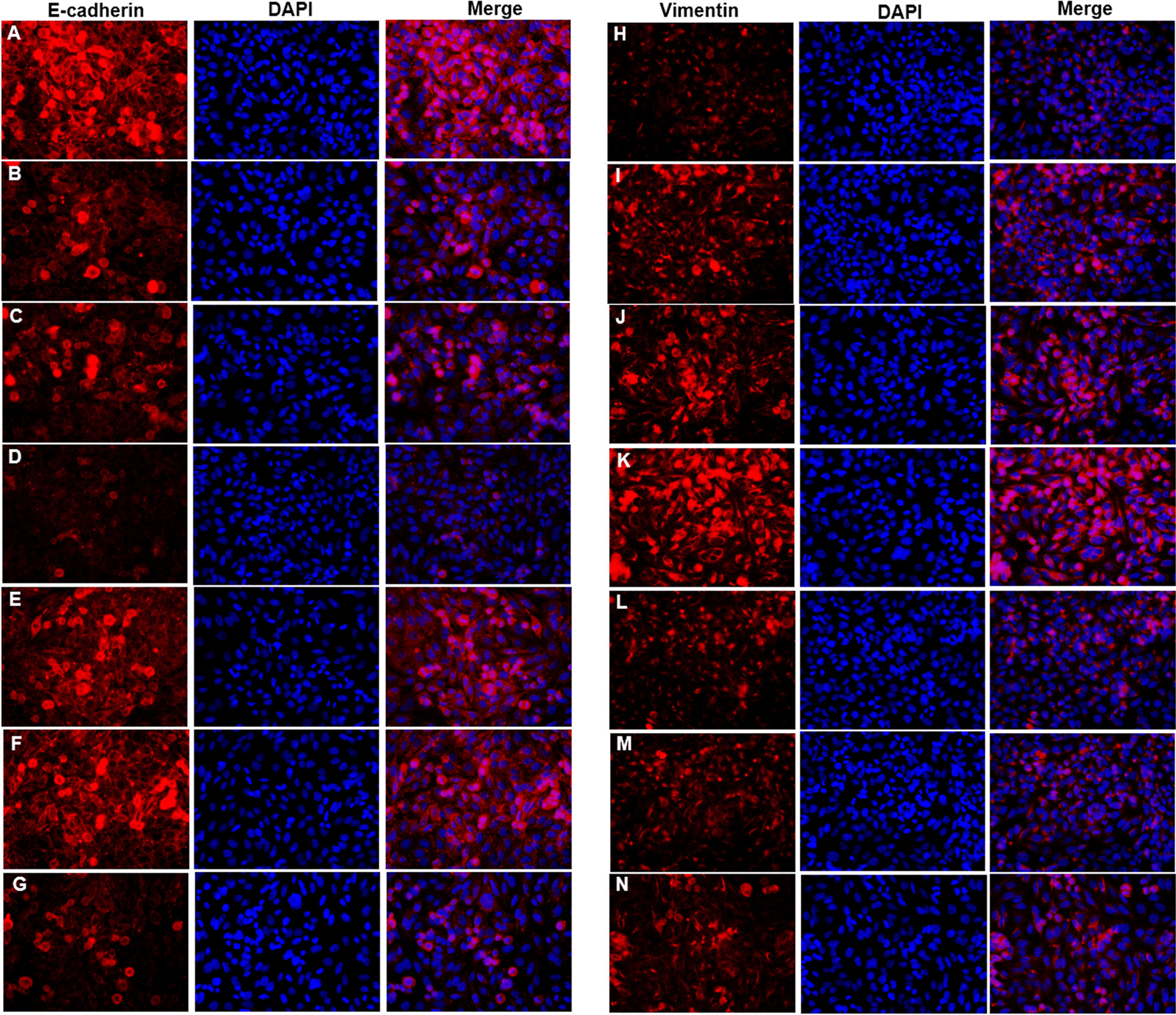
Immunofluorescence staining of E-cadherin and Vimentin in bronchial epithelial cells after silenced C-EBPβ. **a** Control group: E-cadherin. **b** CSE + scramble group: E-cadherin. **c** IL-17A + scramble group: E-cadherin. **d**) CSE + IL-17A + scramble group: E-cadherin. **e** CSE + siRNA group: E-cadherin. **f** IL-17A + siRNA group: E-cadherin. **g** CSE + IL-17A + siRNA group: E-cadherin. **h** Control group: Vimentin. **i** CSE + scramble group: Vimentin. **j** IL-17A + scramble group: Vimentin. **k** CSE + IL-17A + scramble group: Vimentin. **l** CSE + siRNA group: Vimentin. **m** IL-17A + siRNA group: Vimentin. **n** CSE + IL-17A + siRNA group: Vimentin (× 400 magnification)

When C-EBPβ was interfered, the E-cadherin protein expression in CSE group (Fig. 8e), IL-17A group (Fig. 8f) or CSE + IL-17A group (Fig. 8g) was higher than those in siRNA-controls (Fig. 8b–d). In contrast, the Vimentin protein expression in CSE group (Fig. 8l), IL-17A group (Fig. 8m) or CSE + IL-17A group (Fig. 8n) was lower than those in siRNA-controls (Fig. 8i–k). These results indicate that when C-EBPβ is inhibited, the bronchial EMT is reduced. The mRNA expressions of E-Cadherin and Vimentin in bronchial epithelial cells were in consistent with the protein expressions (Fig. 3c, d). Thus, the CSE and IL-17A synergistically induces bronchial EMT through C-EBPβ signaling.Thus, the CSE and IL-17A synergistically induces bronchial EMT through C-EBPβ signaling.

## Discussion

Our study demonstrated an increased expression of IL-17A and C-EBPβ in lung tissues, and an enhanced bronchial EMT of COPD mice. In in vitro study, we further found an increased expression of C-EBPβ and IL-17R in murine bronchial epithelial cells when induced by CSE and IL-17A. When C-EBPβ was inhibited, CSE and IL-17A-induced EMT in murine bronchial epithelial cells was reduced. These results suggest that cigarette and IL-17A could synergistically induce bronchial EMT via C-EBPβ signaling in COPD mice.

In our study, COPD mice had an increased inflammation, extracellular matrix, smooth muscle thickening, goblet cell hyperplasia and mucous secretion in lung tissues when compared with controls. The IL-17+ cells were more expressed in lung tissues of COPD mice than controls. These results suggest that in our model, IL-17 may promote pathological changes of lung in COPD, which consistent with previous findings in lung tissues of COPD patients [6]. Moreover, E-Cadherin distribution was decreased and Vimentin distribution was increased in bronchus of COPD group when compared with controls. And the mRNA expressions of E-Cadherin and Vimentin in lung tissues confirmed those findings. The decrease of E-cadherin expression and increase of Vimentin expression could indicate bronchial EMT [28]. Thus, IL-17 may contribute to bronchial EMT in COPD.

We further investigated the effect of CSE and IL-17A on bronchial EMT with separated murine bronchial epithelial cells. E-Cadherin expression was lowest and Vimentin expression was highest in bronchial epithelial cells when induced by CSE + IL-17A, followed by CSE group and IL-17A group. Those suggest that cigarette smoking and IL-17A could synergistically induce EMT in bronchial epithelial cells. Our in vitro study also showed an increased expression of IL-17R in bronchial epithelial cells when induced by CSE or/and IL-17A, further demonstrating a possible effect of IL-17A and CSE on bronchial epithelial cells.

Moreover, we explored the mechanism how IL-17A and CSE influence the EMT of bronchial epithelial cells. In the present study, the C-EBPβ+ cells in lung tissues of COPD mice distributed more than controls, which was further confirmed by protein expression using western blotting. That was consistent with previous report that C-EBPβ expression in small airway epithelia was increased in COPD, and could be regulated by IL-17A via IL-17R [23, 24]. Thus, the mechanism of IL-17A/ C-EBPβ in airway epithelia cells was further investigated in vitro in our study.

Furthermore, in in vitro study, we found that CSE or IL-17A could up-regulate C-EBPβ protein expression in bronchial epithelial cells, and CSE combined with IL-17A could induce highest expression of C-EBPβ protein. Those findings suggest that C-EBPβ should be the downstream of CSE/IL-17A in bronchial epithelial cells. C-EBPβ activation could be stimulated by CSE combined with IL-17A in bronchial epithelial cells. Interestingly, when C-EBPβ was silenced by siRNA in bronchial epithelial cells, the EMT was decreased when compared with those without C-EBPβ silenced. Those results further confirmed C-EBPβ as the downstream of CSE/IL-17A-induced EMT in bronchial epithelial cells.

In lung cancers, EMT activity was found similar in squamous cell- and adeno-carcinomas [29], and poor patients’ outcomes were correlated with increased EMT [30]. Those findings demonstrate that EMT is an important histopathological process in lung tumorigenesis. Interestingly, EMT of human lung epithelial cells could be medicated by C-EBPβ [31], which also participate tumorigenesis in lung [32]. Thus, C-EBPβ may be a potential target to prevent tumorigenesis of airway in the context of cigarette smoking. Since pathological association of smoking with the development of COPD and then lung cancer may well have links with EMT and its downstream pathways, and COPD itself is a strong risk factor for lung cancer [33], findings from our study may provide a noval target in inhibiting lung cancer developing from COPD.

## Conclusions

In conclusion, C-EBPβ mediates in cigarette/IL-17A-induced bronchial EMT in COPD mice. Our findings contribute to a better understanding on the interaction between COPD and lung cancers, which will provide novel avenues in preventing tumorigenesis of airway in the context of cigarette smoking.

## Data Availability

The datasets used and/or analyzed during the current study are available from the corresponding author on reasonable request.

## Abbreviations

COPD: Chronic obstructive pulmonary disease
C-EBPβ: CCAAT/enhancer-binding protein β
EMT: Epithelial–mesenchymal transition
CSE: Cigarette smoke extract
IL-17R: IL-17 receptor
HE: Hematoxylin and eosin
PAS: Periodic acid–Schiff
